# Valedictory Address to the Graduating Class of the Woman’s Medical College

**Published:** 1882-07

**Authors:** I. N. Danforth

**Affiliations:** Professor of Pathology and Renal Diseases in the Woman’s College; and of Clinical Medicine in the Chicago Medical College


### ARTICLE III.

VALEDICTORY ADDRESS TO THE GRADUATING CLASS OF THE WOMEN'S MEDICAL COLLEGE OF CHICAGO, March, 1881. By I. N. DANFORTH, A.M., M.D.; Professor of Pathology and Renal Diseases in the Woman's College; and of Clinical Medicine in the Chicago Medical College.

LADIES OF THE GRADUATING CLASS: You have each of you now received the degree which you have so long coveted, and for which you have toiled so long and well. For each one of you the past three years have been years of unremitting labor—and for some, perhaps all, of you—they have been years of self-denial as well.

With the rising of to-morrow's sun will come this question: "What next?"—and it is a question so vastly important in its relations to your future lives, that you cannot yet grasp its full meaning.

But it is not by any means a question which interests you alone; it is of hardly less importance to these friends who have favored us by their presence this evening, and in fact it is a question which has a practical interest for every community of our land. Regarded in its broader aspect, the question "what next?" means, not what *you* will do to-morrow or next year; not what shall be the measure of *your* success or the depth of *your* failure; not so much what shall be the outcome of *your* lives, but what shall be the future of woman in the profession of medicine; and it is your proud privilege to be ranked among those to whom the settlement of this question is committed. Without attempting to answer, or indeed to exhaustively discuss the subject this evening, I desire to lay down a few propositions, which I would have you accept as a sort of basis upon which to build your future professional lives.

And, first, I would have you go forward in full realization of the consciousness that you have a perfect *right* to study and practice medicine; that you have not entered upon a life of medical practice because the privilege so to do has been *granted* you, but because you have an inherent and inalienable *right* to that effect. The male portion of the community has no exclusive jurisdiction over the profession of medicine, or the profession of law or of theology, hence they can grant to you no privilege to practice or to preach. The right of woman to earn her daily bread and achieve honorable fame in any and every honorable vocation; comes from God, and cannot be monopolized by man. I say it cannot be monopolized by *man*.

I am aware that the savage tribes enslave their women and make them beasts of burden, but a savage is not a man, as regards his relations to citizenship or civilization. I am also aware that here and there, in civilized countries, and, incredible as it may seem, even in our own republic, we come across an unclassified animal whom, until his place in zoölogy is definitely fixed, we are compelled to call a man, who denies that woman has any *rights* outside of rocking the cradle and mending the stockings. But these apparent exceptions to the fact which I have formulated, rather serve to strengthen than weaken my position, and in practice they benefit, rather than injure woman, in that they unify your sex, and thus vastly increase your strength, and in that they lead every true man to heartily and cheerfully recognize the fact that woman is endowed with inalienable rights, and not merely with privileges which can be granted and revoked at will. Therefore, I say to you, ladies of the graduating class, enter any community you please, and engage in the practice of your profession, because it is your *right*.

In the second place, I present this proposition for your acceptance: that your practice cannot be environed by sex or age. It is very common for us to hear something like this: That it is all very well for women to practice among their own sex and to doctor the babies, but that they can never engage in general practice. But to my mind, nothing is more certain than this: that women *must* engage in general practice, or they will not practice at all, either acceptably or permanently. It is absurd, as well as impossible, to build an impassible wall between the sexes, which is based upon any profession. If a woman practice law, she must practice *all* law; if a woman preach the Gospel, she must preach it to all; if a woman practice medicine, she must minister to the wants of *all*. The family physician must attend to all the ordinary medical wants of that family, or else there must and will be a change of doctors in that family. And this is not theory merely; experience, in a limited sense, has already demonstrated my position.

Of course, cases will arise, as cases *now arise*, in which the aid of "specialists" or those specially trained in certain departments of practice will be called, but the *fact* will remain that the family physician, be he man, or be she woman, will be the family physician still. And then we shall all wonder that we ever doubted it. Who does not remember the cynical critics who used to frown upon the woman who dared to speak in public? but at the present time women are recognized not only as acceptable, but most effective and useful public speakers.

In the next place, I would have you enter practice with the idea that your services are worth just as much as though you were men. It is a blot upon our civilization, and an offense to our sense of justice and equity, that we attach less pecuniary value to the services of women than of men. Services of all kinds should be paid for according to their real value, not according to sex of the person who happens to render them. Nevertheless, as a general rule, women are paid less than men for the same service. Female clerks, female teachers, female copyists, female telegraph operators are paid less than males in like capacity, and if you extend your range of observation, you will find the same fact true. Indeed, I have heard of one man in this city who asked for a reduction on a doctor's bill, because the doctor who rendered the service was a woman. But such moral malformations do not often arise.

Now, ladies, I would have you enter practice with the full assurance that the value of your services does not depend on your sex, but upon your skill, and remember this: that no one will be likely to value your services higher than you value them yourselves. Not that I would have you become extortioners in the slightest degree, but I would have you thoroughly imbued with a reasonable self-respect, and this is the surest warrant for the outgrowth of a wise and tender pity for suffering humanity.

Again, you must expect to assume all the responsibilities incident to the practice of medicine. Responsibility in medical practice knows no sex ; it sits with equal weight upon all—and this is right. Your own clients, as well as the general public, the medical profession and the press, both secular and professional, will hold you to precisely the same accountability as they hold all other physicians. Community holds all physicians to a very strict account, and community is very sensitive on this point. I am glad that it is so. It adds dignity and honor to our profession ; it ennobles our calling in the eyes of community, and thus, of course, ennobles us. And the people will soon come to regard you as physicians ; not as women merely—precisely as they regard *us* as physicians—and not as men merely.

So, too, the law will hold you to the same measure of legal accountability as I am held, and for exactly the same reason. A suit for malpractice will mean the same for you as it means for me, and the judge on the bench will charge the jury that your legal liabilites are neither more nor less because you are women.

Finally, you are to take your places in the ranks of medicine as *physicians, not* as women ; as exponents of medical science, not as the representatives of sex.

And now, ladies of the graduating class, what does the future promise ? You have completed the studies required by our college ; you have passed our examinations, most creditably to yourselves ; you have received from us the parchment certificate, constituting you physicians, so far as it is in our power to make you such ; and now what does the future promise ? I answer, success. More than this, I guarantee you success. But you ask "When ?" I answer, as soon as you merit it, and if you never merit success, you will, of course, never reach it.

Nevertheless, I expect, and my colleagues now here present expect, success from each one of you. Not that we expect a whole community to send for you to-morrow, or next week, or next year, but we expect you to conquer success by patient, persevering effort ; we expect you to be diligent students, good thinkers and patient waiters. Every young physician has to fairly tire a community out by patient waiting. You will sometimes hear of the wonderful success and the princely income of some pushing young physician who "rode" himself into practice by fast driving, and by other misleading exploits, such as employing some-one to call him from church or some other public assembly, all of which were designed to convince the public that he was flooded with business. Such stories one almost always find pure fictions, utterly without a basis of truth. But whenever temporary success has been achieved by such dishonorable means, it has cost more than it was worth, and the ultimate result has been a legacy of unpaid bills and the well deserved reputation of being sadly deficient in common sense.

Let those who would follow in the footsteps of Mr. Bob Sawyer and Mr. Ben Allen in this particular characteristic, remember that Dickens was always conspicuously successful in the creation of his fools, and never more so than when his magic wand caused these graceless wanderers to posture before him.

The winter has come and gone; lectures are no more; the college building is closed; the lecture rooms are desolate; never again shall *we*—*this* class and *this* faculty—gather in the old familiar places. To-morrow you will glide away, as fast as steam can hurry you, to your respective homes, there to receive the well-earned plaudits and affectionate greetings from your friends and relatives. A few days hence, when the visits are all made and the congratulations are all in and you have rested from your winter's work, time will begin to hang heavily, the reaction will come and the stern, hard, cold question, "what next?" will be upon you, in all its inexorable reality. Do not spend one hour in doubting or hesitating; move forward to your place in the world; commence life in earnest; assume at once and gladly the grave responsibilities for which you have prepared yourselves. Show that you have confidence in yourselves; it is the first—and possibly the most important—step toward a successful professional life. Welcome the coming spring, so soon to burst upon us, with smiling faces, and hearts full of gratitude, that the world recognizes you among its youthful workers, and then go bravely and patiently to occupy and adorn your appointed place.

It only remains for me to assure you, ladies of the graduating class, that the faculty will rejoice in your future growth and will watch your professional developments and unfoldings, with all the interest which a tender Alma Mater ought to feel in her growing daughters; and, on behalf of my colleagues, as well as myself, to pronounce the word which you have so ardently longed to hear, and yet which at this moment we all utter with sadness—Farewell.

